# Anaesthesia for Renal Transplantation: An Update

**Published:** 2009-04

**Authors:** Vaibhavi Baxi, Anand Jain, D Dasgupta

**Affiliations:** 1 Clinical Associate, Jaslok Hospital and Research Center, Mumbai; 2 Clinical Associate, Jaslok Hospital and Research Center, Mumbai; 3 Head of the Department of Anaesthesia, Jaslok Hospital and Research Center, Mumbai

**Keywords:** Renal transplantation, Anaesthesia management, Recipient, Cadaver donor, Living donor

## Abstract

**Summary:**

Attempts at organ transplantation have been made since the 19^th^ century. Renal transplantation is the preferred treatment for end stage renal disease. Renal transplant anaesthesia requires a thorough understanding of the metabolic and systemic abnormalities in end stage renal disease, familiarity with transplant medicine and expertise in managing and optimizing these patients for the best possible outcome. Also, the associated co-morbid conditions increase the complexity of anaesthesia, pain management and perioperative morbidity and mortality. Hence, a good perioperative management of these patients includes a multidisciplinary collaboration with well-planned anaesthetic strategies.

## Introduction

Successful transplantation to replace failed human organs was a daunting goal at the start of the 20th century. Investigators in Vienna initially attempted kidney transplantation in several animals in 1901. After 5 decades, Dr. René Küss performed the first kidney transplantation that functioned in humans. The kidney worked without immunosuppression but was rejected 2 months later. It was in 1954 that Dr. Joseph Murray performed the first successful kidney transplantation using a kidney from an identical twin. Further progress was made with advances in immunosuppression—the use of azathioprine in 1959 by Dr. Roy Calne and its combination with steroids by Dr. Thomas Starzl. The introduction of antilymphocyte globulin by Dr. Starzl in 1967 and development of organ preservation solutions by Dr. Folkert Belzer (1968) and Dr. Jeffery Collins (1969) enabled the use of allografts from remote organ donors and better outcome as compared to earlier transplants.^1^

## Clinical problems in renal failure related to anaesthesia

The kidneys are essential for adjusting body fluid volumes, electrolyte composition, acid base balance and hemoglobin concentration. They receive about 25% of cardiac output and function as filters for toxins and drugs in the circulation. Chronic renal failure or more appropriately chronic kidney disease (CKD) refers to a decline in the glomerular filtration rate (GFR) caused by a variety of diseases such as diabetes mellitus (40%), hypertension (27%), chronic glomerulonephritis (13%), cystic kidney disease (3.5%), interstitial nephritis (4%) and other diseases such as obstructive uropathy, lupus nephritis and human immunodeficiency virus.^2^ CKD may be categorized as mild (GFR of 60-89 mL/min/1.73 m^2^), moderate (GFR of 30-59 mL/min/1.73 m^2^), severe (GFR of 15-29 mL/min/1.73 m^2^), or end-stage renal disease (ESRD). Hemodialysis or peritoneal dialysis is typically initiated as the GFR falls to less than 15 mL/min/1.73 m^2^. The progression of renal disease from one stage to the next results in deleterious effects on multiple organ systems.^3^

### Cardiovascular system

Almost 50% of deaths in patients with CKD are due to involvement of the cardiovascular system. Damage starts in early stages and frequently in the form of IHD, dilated cardiomyopathy, CCF, LVH and pulmonary hypertension. Accelerated arteriosclerosis is promoted by diabetes and dyslipidemias, while hypertension and cardiomyopathy is usually due to both volume and pressure overload and high levels of renin-angiotensin. Volume overload occurs due to expansion of ECF, high blood flow through AV fistulae and anemia, while pressure overload is due to hypertension. Administration of erythropoietin for improving haemopoiesis may further raise the blood pressure and increase the requirement of antihypertensive drugs. The goal is to achieve a blood pressure of <130/85 mm Hg. Occasionally, uremic pericarditis of the hemorrhagic type may be seen that may progress to cardiac tam-ponade. It is less often seen now because dialysis is started before it appears.^4^

### Hematological system

Normochromic, normocytic anemia occurs due to impaired erythopoiesis secondary to decreased erythropoietin synthesis and release, decreased red cell life span, increased hemolysis and bleeding, repeated loss during hemodialysis, aluminum toxicity, uremia induced bone marrow suppression and iron, folate and vitamin B_6_ and B_12_ deficiencies. These patients may have haemoglobin levels of 5 to 7 g/dl (hematocrit of 15-25%). Compensatory mechanisms to overcome the decrease in oxygen carrying capacity include an increase in cardiac output and 2,3-DPG causing a right shift of oxygen dissociation curve and thus improving tissue oxygenation. Use of biosynthetic erythropoietin and darbopoietin is associated with increase in Hb and reduced need for repeated blood transfusions, which decreases the risk of sensitization.^5^ Although the beneficial role of transfusion is controversial in cyclosporine era, there need be no hesitation in replacing volume losses with packed, washed and irradiated red blood cells, keeping in mind that this may lead to an increase in plasma potassium levels.^6^

### Respiratory System

Pulmonary congestion due to volume overload results in hypoxemia and hypocapnia. Intraperitoneal fluid used in peritoneal dialysis can cause diaphragmatic splinting with basal atelectasis and shunting. Uraemic lung is a radiological entity characterized by perihilar congestion.

### Electrolytes and Acid Base Status

Inability to excrete water, electrolytes and free acids results in metabolic acidosis, hyponatremia, hyperchloremia and hyperkalemia. For every 0.1 unit change in pH, potassium increases by 0.6 mEq/L. Severe hyperkalemia increases cardiac and skeletal muscle excitability. The ECG shows peaked T waves, flat P waves, increased PR interval and a wide QRS complex that can progress to sine wave and ventricular fibrillation. Treatment involves use of 10 ml of 10% calcium gluconate iv, 1 mEq/Kg sodium bicarbonate iv, β agonists, hyperventilation in mechanically ventilated patients, furosemide and magnesium. However, hemodialysis or peritoneal dialysis is the definite treatment. Hypermagnesemia usually accompanies hyperkalemia (GFR < 10 ml/minute) and can cause neuromuscular weakness, respiratory failure, bradycardia, hypotension and heart block.^2^,^7^

### Endocrine System

As GFR falls, phosphate excretion falls leading to reduced absorption of calcium from gastrointestinal tract and vitamin D deficiency. Hyperactivity of parathyroid glands attempts to maintain calcium. This secondary hyperparathyroidism however leads to osteomalacia, osteosclerosis and osteitis fibrosa cystica culminating into a clinical entity known widely as uremic osteodystrophy. The result is bone demineralization making these patients susceptible to spontaneous pathological fractures.^8^

### Coagulation

Accumulation of endogenous toxic products like guanininosuccinate, phenol and phenolic acids leads to platelet dysfunction and decreased levels of platelet factor III. PT and PTT remain normal but bleeding time is prolonged. Treatment includes platelet transfusion, cryoprecipitate, desmopressin acetate or conjugated estrogen.^9^

### Central Nervous System

The manifestations include malaise, fatigue and inability to concentrate, pruritus progressing to myoclonus, seizures, coma and death. Dialysis dysequilibrium syndrome resulting from changes in ECF volume, electrolyte composition and cerebral edema is characterized by dehydration, vomiting and hypotension. Dementia affects patients on long-term dialysis and may be due to aluminum toxicity.

### Gastrointestinal System

Anorexia, nausea, vomiting, gut bleeding and diarrhea and hiccups are common. Delayed gastric emptying time, increased acidity and gastric volume necessitate use of H_2_ blockers and proton pump inhibitors.

### Problems of Dialysis

Main sequel are excessive or persistent heparinization, abnormal fluid shifts, β_2_ microglobulinemia, dialysis dysequilibrium syndrome, hepatitis, HIV, leucopenia and hypocomplementemia.^10^ Also, poor care of AV fistulae can lead to local gangrene, sepsis and the need for amputation of the limb. Peritoneal dialysis on the other hand can cause peritonitis and sub-acute intestinal obstruction.

### Influence of renal failure on pharmacokinetics and pharmacodynamics of anaesthetic agents

Lipid soluble, unionized drugs are extensively reabsorbed by renal tubular cells. Termination of their action is not dependent on renal excretion. After biotransformation these drugs are excreted as water soluble, polar forms of the parent compound. Lipid insoluble or highly ionized drugs in the physiologic range are eliminated in urine. Their duration of action may be extended in patients with impaired renal function.^10^

### Premedication Drugs-

Atropine and glycopyrrolate are eliminated 20-50% by kidney. Because they are administered as single doses accumulation with toxic effects is unlikely to be a significant problem. H_2_-histamine receptor antagonists such as ranitidine, famotidine are largely unaltered by end stage renal disease. With metoclopromide (<20% elimination) there is significant reduction in clearance (16.7 L/h compared with 52.5 L/h) and prolongation of the terminal half life (13.9h compared with 2.8h).^11^

With benzodiazepines there is decrease in the plasma protein binding, increased volume of distribution and increased systemic clearance secondary to increased free unbound fraction (1.4% to 7.9%) of diazepam in patients with CKD.^12^ CKD does not alter the distribution, elimination, or clearance of unbound midazolam. Changes in the pharmacodynamic profile of midazolam in CKD patients, if they exist, are more likely due to inherent alterations in drug sensitivity than to pharmacokinetic changes.^13^ After a single oral dose (2.5 mg) the biotransformation of lorazepam to its glucuronide conjugate remains unaltered. Urinary excretion of lorazepam-glucuronide considerably decreases in chronic renal failure associated with accumulation of high concentrations of this conjugate in plasma during days after a single oral dose.^14^

### Induction Agents-

Low serum albumin levels leads to an increase in free fraction of the drug in plasma while uremia associated altered blood brain barrier can increase the levels of unbound drug crossing the blood brain barrier into CNS receptors. Hence, the dose of induction agents may need to be adjusted according to the volume status, acidic pH and increased sensitivity of the nervous system to these drugs.

In chronic renal failure patients, the underlying rate and extent of thiopental distribution and elimination are much the same as in normal patients.^15^ However, a higher dose of propofol is required to reach the clinical end point of hypnosis and bispectral index of 50. The hyperdynamic circulation and high plasma volume resulting from anemia can counteract the effect of low serum albumin explaining the higher dose requirement with propofol.^16^ Ketamine pharmacokinetics are not significantly changed by renal disease, but the hypertensive effects make it undesirable in patients with underlying hypertension. Etomidate is well tolerated and preserves hemodynamic stability. The associated steroid suppression is short-lived and is of little relevance in transplant patients concurrently receiving hydrocortisone for immunosupression.

### Opioids-

Morphine is metabolized in liver to morphine-6-glucuronide (M6G), morphine-3-glucuronide (M3G) and normorphine, all of which are excreted by the kid-neys. M6G accumulates in renal failure and mediates CNS and respiratory depression. Meperidine is metabolized in liver to normeperidine, also excreted by kidneys. Accumulation of these metabolic products leads to excitatory CNS effects such as convulsions. Fentanyl is metabolized by the liver with only 7% excreted unchanged in urine making it suitable and safe for short-term use during surgery. However, if used for long duration, the pharmacodynamic effects should be monitored in view of parent compound accumulation. The clearance and half-life of sufentanil are not significantly altered in patients with reduced renal function. Remifentanil is mainly metabolized by blood and tissue esterases while its principle metabolite is eliminated by kidney. Reduced elimination of this metabolite is not of clinical significance because of its low potency - 1/4000 of its parent compound.^17^

### Muscle Relaxants-

Succinylcholine can be used in patients with renal failure provided potassium concentration is less than 5.5 mEq/l and repeated doses are avoided. Plasma cholinesterase has been reported to be below normal in more than 20% of end stage renal disease patients whether they are receiving any form of dialysis or not.^18^

Prolonged duration of action of non-depolarizing agents is primarily due to delayed clearance.

**Table:** 

Drug	Renal excretion	Normal t1/2	Anephric t1/2
Pancuronium	85%	132 min	258min
Rocuronium	10%	42min	58min
Vecuronium	40-50%	54min	84min
Atracurium	10-40%	18min	24min
Cisatracurium	16%	34min	No effect
Mivacurium	<5%	1.8min	3.6min
Succinylcholine	<25%	1min	1min

### Volatile Anaesthetics –

Sevoflurane and enflurane undergo biodegradation to inorganic fluoride. A serum fluoride concentration of 50micro-moles/l is the peak value that is nephrotoxic. There is evidence of transient impairment of renal concentrating ability and renal tubular injury in patients receiving sevoflurane and enflurane.^19^ FDA recommends the use of sevoflurane with fresh gas flows rates atleast 1L/min for exposures up to 1hr and atleast 2 L/min for exposures greater than 1 hr.

Fluoride levels after isoflurane and halothane increase by 3-5 micro-moles/Land 1-2 micro-moles/L, respectively. Hence, the risk of nephrotoxicity is remote. Desflurane is resistant to biodegradation and so even a prolonged exposure to desflurane (7.0 MAC hrs) has been associated with normal renal function.

### Anticholinesterase Drugs –

Renal excretion accounts for approximately 50% of the clearance of neostigmine and approximately 75% of elimination of edrophonium and pyridostigmine. Renal failure allows some protection against residual NM blockade because renal elimination half times of anticholinesterase drugs is prolonged.^6^

### Organ Matching and Allocation^20^

The sources of donor kidney can either be a living related donor, living unrelated donor or a deceased cadaveric donor. Initial testing is done to determine major blood group compatibility. Cadaveric kidney should be ABO identical to the recipient while a live donor kidney may be either ABO identical or compatible. However, it is possible to place an ABO-incompatible organ in a recipient using various protocols like plasmapheresis and immunoabsorption to overcome rejection as the Rh system is not expressed in the graft tissue.

The human major histocompatibility complex is a cluster of genes on chromosome 6 that encode human leukocyte antigens (HLA). Before transplant, HLA antigens are identified by DNA based methods in all donors and recipients. Outcome is best with a perfect HLA matched donor and recipient. A crossmatching test determines if the recipient has serum antibodies directed against donor HLA lymphocyte antigens. These may arise as a result of exposure through pregnancies or blood transfusions in past. A positive crossmatch may cause rejection of graft. Currently, the methods to reduce allosensitization are use of immunoglobulins and plasmapheresis.

### Immunosupression -

Different centers in the world use several regimens of immunosupressants to decrease the incidence of graft rejection. Their use is divided in three phases.

The first phase is induction therapy started before and during first week post transplant and involves marked immune suppression. Induction agents are-Thymoglobulin, OKT3, Daclizumab, or Basiliximab.

The second phase is maintenance therapy involving drug administration continuously for three to six months to prevent acute graft rejection and induce tolerance.

The third phase involves long-term immunosupression maintained for the rest of the life.

The follwing drugs are used for immunosupression –21

* Steroids

* Calcineurin inhibitors (CNI)

Cyclosporin

Tacrolimus

* Target of rapamycin(TOR)inhibitors

Sirolimus

Everolimus

* Polyclonal antibodies

Antilymphocyte globulin

* Monoclonal antibodies

Interleukin 2

Daclizumab

Basiliximab

OKT 3

* Purine synthesis inhibitors

Azathioprine.

Interaction of these agents with anaesthetic drugs is not clinically significant.

### Donor kidney -

The number of living donors has exceeded that of cadaver donors because of low morbidity after living kidney donation and availability of minimally invasive approach like laparoscopic donor nephrectomy and the recent robotic hand assisted donor nephrectomy.^22^ The traditional approach though is a subcostal lateral incision. The left kidney is preferred because of better surgical exposure and longer vascular supply. Position is lateral with table flexed and kidney rest elevated. One or two large peripheral venous lines usually suffice and invasive monitoring is not required. To maintain good diuresis, fluid administration is generous (10-20ml/kg/hr) using isotonic crystalloids intraoperatively. In fact, some centers recommend overnight preoperative hydration with intravenous fluids and preloading the patients with colloids just before induction of anaesthesia. Loop diuretics and/or mannitol may be used to promote diuresis from the grafted kidney. Mannitol improves renal blood flow, acts as a free radical scavenger and reduces the incidence of impaired renal function immediately after transplant. Intravenous Heparin 2000-5000 IU may be given before clamping the renal vessels. Postoperative pain is usually mild to moderate and managed in most cases using intravenous opioids. Deceased donor kidneys are preserved using hypothermia and pharmacologic inhibition to slow down metabolic process. Cold storage solutions are agents that do not readily permeate the cell membrane and have an electrolyte composition similar to the intracellular environment (low sodium, high potassium), there by preventing loss of cellular potassium. Cold ishaemia times should be kept below 36-40 hours in case of cadaveric donors after which the incidence of delayed graft function increases significantly. Transfer in a pulsatile preservative solution machine improves organ viability.^23^ Kidney from living donor may be flushed with preservative solution or iced Ringer's lactate solution containing heparin and mannitol. The cold ishaemia time in a living donor should be restricted to 20-30 minutes while the warm ischemia time should not exceed 3-5 minutes.

## Anaesthetic Management of Renal Transplant Recipient

### Preoperative considerations-

Recipients involving cadaveric donor organs are often scheduled as urgent or emergency procedures. However, a well-preserved kidney provides enough time to prepare the recipient and if necessary dialyze to normalize electrolyte and volume imbalance. The recipient should have routine systemic tests such as CBC, platelet count, electrolytes, serum glucose, BUN, serum creatinine, PT, PTT, INR, liver function tests, urinanalysis, ECG, chest radiograph and 2D Echocardiogram. Evaluation of cardiac function is of central importance. To help detect coronary artery disease and perhaps to lower the risk of adverse effects with transplantation, all patients and especially diabetic patients with ESRD are evaluated for the presence and/or absence of coronary artery disease. Besides routine ECG, echocardiogram and treadmill test, dobutamine stress echocardiogram is being increasingly used as the initial noninvasive test, given the superiority of this test compared with other examinations and the potential adverse effects of catheterization. Dialysis if indicated is done within 24 hrs of the operation. Overzealous ultrafiltration is best avoided. Volume status is roughly estimated by their dry weight. Loss of more than 2kg during dialysis suggests significant intravascular depletion.^24^ Antihypertensives drugs should be continued until the time of surgery. Oral hypoglycemic agents should be held on the morning of the surgery. Sliding scale insulin regimen may be used intraoperatively if blood glucose levels are high. Antibiotic prophylaxis includes a first generation cephalosporin or if penicillin allergic, vancomycin. Induction of immunosupression is started before entering operating theatre.

### Intraoperative management-

Successful use of regional anaesthesia has been reported by some centers.^25^,^26^ Certain factors to be considered for the use of regional anaesthesia areuraemic bleeding tendency, effect of residual heparin given during dialysis, altered platelet function, decrease in coagulation factors and the duration of surgery. The advantages of combined spinal-epidural technique arerapid onset and good muscle relaxation from spinal and supplemental analgesia through epidural during and after surgery. Most centers however use balanced general anaesthesia to provide stable hemodynamics, excellent muscle relaxation and predictable depth of anaesthesia.

Standard ASA monitors are adequate, although, patients with more advanced co-morbid conditions require extensive monitoring such as continuous arterial pressure or CVP monitoring.^27^ Those with the most severe co-morbid conditions, such as symptomatic CAD or history of congestive heart failure, should be monitored with a pulmonary artery catheter or transesophageal echocardiography. Strict asepsis should be maintained at all times.

The status of hemodialysis shunts or fistulae should be monitored during positioning and intraoperatively. Assesment of the area over the fistula for infection, redness, edema, soreness, warmth and palpation of distal pulses should be routinely done. To establish and document the patency of the fistula, palpation for a thrill or vibration and auscultation over the fistula for a swishing noise or bruit is mandatory. The fistula may have to be covered with soft gamgee rolls to prevent any trauma intraoperatively. Also, great care should be taken while transferring these patients on to the operation table as they are prone to patholological fractures.

Risk of aspiration during induction of anaesthesia necessitates rapid sequence induction while maintaining cricoid pressure. The induction drugs should be given slowly to minimize drug-induced hypotension. Attenuation of sympathetic nervous system by antihypertensives, diabetic autonomic neuropathy, disruption of blood brain barrier, increased levels of unbound drug and increased sensitivity of central nervous system makes the patient vulnerable to hypotension on induction. Propofol, thiopentone, or etomidate can all be used in routine circumstances. Patients with preexisting hypertension and underlying coronary artery disease are at high risk of large fluctuations in heart rate and blood pressure during induction and intubation. Short acting beta adrenergic blocker esmolol and short acting opioids like fentanyl, remifentanil have been effective for blunting the hemodynamic response to intubation. Succinylcholine can be safely used in patients with chronic renal failure. If preoperative potassium is high normal or if there is underlying metabolic acidosis, additional increase of 0.5 to 1.0 mEq per L may occur with succinylcholine administration and hence should be avoided. When choosing a non depolarizing agent for maintenance, it is better to use ones that are independent of renal clearance mechanisms (cisatracurium, atracurium, mivacurium). The choice of inhaled anaesthetic includes desflurane, isoflurane and sevoflurane. The metabolism of sevoflurane has been implicated in renal toxicity with production of fluoride ions and compound A formed by breakdown of sevoflurane by sodium or barium hydroxide. However, studies have shown that fresh gas flows more that 4L/ min did not change renal function indices.^28^ Fentanyl, sufentanil, alfentanil and remifentanil are suitable for perioperative pain control, while morphine and pethidine are best avoided.

Postdialysis patients have intravascular volume depletion. To decrease the incidence of postoperative acute tubular necrosis, a liberal hydration policy is employed intraoperatively. The systolic blood pressure is maintained between 130-160 mm of Hg, CVP between 10-15 mm of Hg and mean pulmonary artery pressure of 18-20 mm of Hg to optimize cardiac output and renal blood flow.^29^ Crystalloids solutions are usually preferred to correct fluid and electrolyte imbalance, however in situations of severe hypovolemia, colloids may be used. Over the last few decades, there has been a shift in practice from using natural colloids such as blood, albumin and fresh frozen plasma to synthetic colloids. Most anaesthesiologists avoid potassium-containing fluids during renal transplantation with the belief that it may worsen hyperkalemia in case of impaired graft function. Balanced crystalloids should be alternated with normal saline (0.9%) as large volumes of saline could lead to hypercholraemic acidosis. Hypotension may occur after unclamping the iliac vessels and reperfusion of the graft. It is critical that patient is well hydrated, as renal function is critically dependent on renal perfusion. It is especially important in paediatric recipients because reperfusion of an adult size kidney graft may divert a significant amount of their own blood volume. Vasopressors with alpha agonist activity should be avoided as they can compromise blood flow to the transplanted organ.^30^ Immediate graft function has been associated with a blood volume greater than 70ml/kg and a plasma volume greater than 45ml/kg.^31^ CVP may decline 25%-50% 1-2 hrs after revascularisation despite aggressive fluid management. This decline is similar in recipients of cadaveric as well as living related donor kidney and the cause may be multifactorial such as redistribution of fluids, changes in vascular permeability or increased nitric oxide levels. Increased hydration works by atrial distention and subsequent release of atrial natriuretic peptide and increased renal perfusion. Transfusion when required should be preferably with packed cells that are saline washed, irradiated and cytomegalovirus negative.

Immediate urine production is seen in over 90% of living donor kidney and between 40%-70% of cadaveric transplants. A decrease in urine production at the latter stages of closure of surgical wound, a decrease in urine output strongly suggests mechanical impingement of the graft, vessel or ureter. The urinary catheter should be irrigated to ensure that clot or tissue has not affected its patency. If intraoperative ultrasound is available, it can be used to examine the flow in arterial and venous anastomosis. Loop diuretics, mannitol and occasionally dopamine may be used to enhance urine production. Mannitol induces osmotic diuresis and also has a protective effect on the tubular cells of the transplanted kidney from ishaemic injury. Loop diuretics block the Na/K channels present in the thin ascending limb of Henle, while low dose dopamine is commonly used to stimulate DA_1_ dopaminergic receptors in the kidney vasculature to induce vasodilatation and increased urine output. However, the utility of this approach is questioned in that a newly transplanted, denervated kidney may not respond to low dose dopamine like normal kidneys do.^32^

### Postoperative Care-

Renal transplant recipients should be reversed and extubated once the established criterion for extubation is fulfilled and there is no concern for airway protection. In general, renal transplant patients are postoperatively nursed in a high-dependency unit. They rarely require intensive care unit admission unless there is fluid overload, a cardiac event or sepsis. Strict monitoring of fluid input output is essential. Re-exploration of the wound should not be delayed if kinking of the vascular attachments or obstruction of the ureter along its course is suspected.

Postoperative pain is usually mild to moderate after renal transplant. Patient controlled analgesia with opioids and intercostals nerve blocks have been successfully used.^33^ Non-steroidal anti-inflammatory drugs inhibit prostaglandins synthesis which are integral for renal blood flow and glomerular filtration rate autoregulation. Hence, these drugs are absolutely contra indicated.

### Anaesthetic Complications After Renal Transplant-

The major postoperative anaesthetic complications are vomiting and pulmonary inhalation, delayed respiratory depression, pulmonary edema, hypotension, hypertension and cardiac arrhythmias which can lead to cardiac arrest. Cardiovascular complications are responsible for 33% of all mortality with 50% showing arterial hypertension.^34^ Factors that lead to increased risk in recipients include age greater than 60 years, coronary artery disease and diabetes mellitus.

## Conclusion

While thousands remain on the waiting list to receive a cadaveric kidney, living-related renal transplantation offers a shorter waiting period and greater survival. Optimizing the health of the recipient before transplantation can improve the ultimate outcome. Effective and safe anaesthesia for renal transplantation patient depends on an understanding of the pathophysiology and biochemistry of uremia and its effect on pharmacokinetics and metabolism of drugs used. Maintaining intraoperative blood volume and the recent improvements in combined immnosuppression therapy have improved the overall outcome in transplant recipients.

As the criterion for accepting patients into renal transplantation programme broadens, the anaesthesiologist is likely to be faced with increasing problems of the interaction of other intercurrent diseases and multiple drug therapies in the near future.
